# The Multifaceted Roles of Diet, Microbes, and Metabolites in Cancer

**DOI:** 10.3390/cancers13040767

**Published:** 2021-02-12

**Authors:** Heather Armstrong, Michael Bording-Jorgensen, Eytan Wine

**Affiliations:** 1 CEGIIR, University of Alberta, Edmonton, AB T6G 2X8, Canada; bordingj@ualberta.ca; 2 Department of Pediatrics, University of Alberta, Edmonton, AB T6G 1C9, Canada; 3 Department of Physiology, University of Alberta, Edmonton, AB T6G 1C9, Canada

**Keywords:** cancer, diet, nutrition, microbes, misconceptions, risk factors, prevention

## Abstract

**Simple Summary:**

The involvement of microbes (virus, fungi, bacteria) and diet in different cancers is slowly being uncovered, yet the complexity of the relationship between these factors has reduced the impact of potential interventions in the clinic. In this review we have highlighted the results of the most recent studies published and have related what the evidence suggests and how we can utilize this knowledge best in directing patients’ diets and future research at this time.

**Abstract:**

Many studies performed to date have implicated select microbes and dietary factors in a variety of cancers, yet the complexity of both these diseases and the relationship between these factors has limited the ability to translate findings into therapies and preventative guidelines. Here we begin by discussing recently published studies relating to dietary factors, such as vitamins and chemical compounds used as ingredients, and their contribution to cancer development. We further review recent studies, which display evidence of the microbial-diet interaction in the context of cancer. The field continues to advance our understanding of the development of select cancers and how dietary factors are related to the development, prevention, and treatment of these cancers. Finally, we highlight the science available in the discussion of common misconceptions with regards to cancer and diet. We conclude this review with thoughts on where we believe future research should focus in order to provide the greatest impact towards human health and preventative medicine.

## 1. Introduction

Although cancer development is known to be complex and related, in many cases, to a diverse array of factors (genetic and environmental), with well-described impacts of longitudinal exposures, there are still critical gaps in the knowledge of what some of these exposures are and how they impact cancer biology. Diet has long been considered a critical and, importantly, modifiable factor in many different biological processes, including cancer. Recent advances in measuring dietary intake and its effect on biological processes (e.g., using metabolomics), and especially the interaction between diet and gut microbes, have opened the way to a myriad of papers implicating diet, the gut microbiome, and related metabolites in the pathogenesis, course, and response to therapy of major cancers. A better definition of how diet is related to cancer biology is especially attractive, as diet is rarely utilized in cancer therapy or prevention and it can provide a safe alternative for intervention in a variety of other chronic conditions [1,2,3,4,5]. However, the potential for diet to affect cancer development and progression remains to be fully elucidated. We focus on diet–microbiome interactions in this setting, as not only do dietary factors and nutrition have profound effects on the health of host cells, but they also affect the human microbiome [6], which in turn is closely linked to cancer.

**Figure 1:**
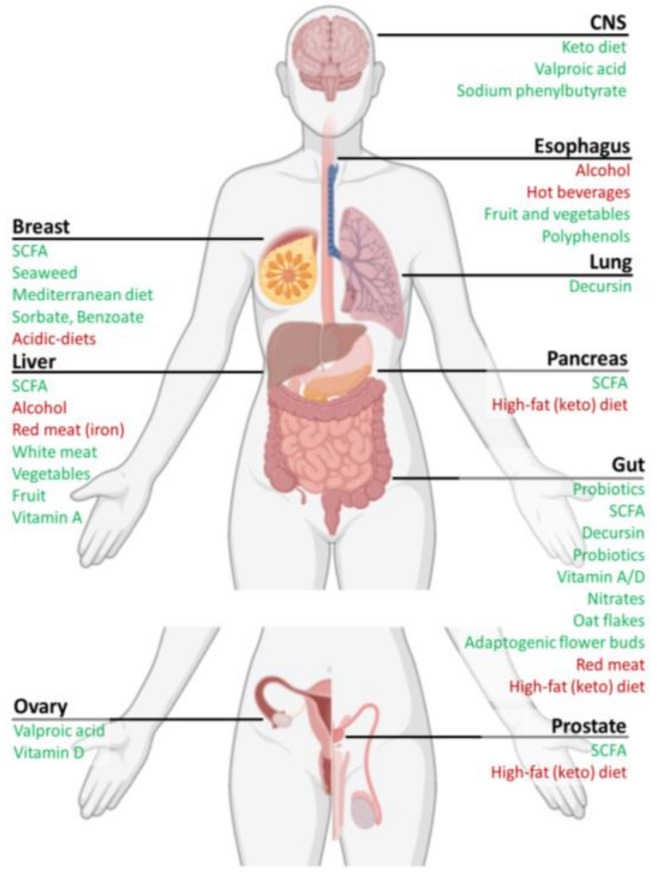
Cancer promoting and preventative interactions of dietary factors and organ systems. Intake of dietary factors, as demonstrated only by more recent research studies, can affect cancer progression through positive (green) or negative (red) effects on organ systems, as indicated. Details are further highlighted in Table 1.

These observations are especially relevant to the gut: emerging evidence continues to demonstrate that nutritional states directly drive intestinal adaptation, resulting in altered signalling mechanisms within adult intestinal stem cells relevant to intestinal tumour formation [7,8,9,10]. Some correlations to chronic conditions, such as inflammatory diseases, may help inspire future research. The link between inflammation and concomitant tumour development in cancers was first suggested by Virchow in 1863 [11,12], and today roughly 20% of cancers are thought to directly result from chronic inflammation [13]. Likely one of the most well studied examples to date is the link between inflammatory bowel diseases (IBD) and colorectal cancer (CRC) [14,15,16,17]. Notably, select dietary factors including saturated fats, red meat, and refined carbohydrates have been suggested to display pro-inflammatory properties and diet has been shown to play a key role in up to 40% of all cancers [18,19,20,21]. These dietary factors are further involved in modulating the gut microbiome, which in turn is involved in regulating both gut physiology and immune response [22,23,24]. This model of IBD−CRC has further demonstrated the critical role of microbes in inflammatory pathways due to their ability to modulate inflammatory cytokines (e.g., tumour necrosis factor (TNF), interleukin (IL)-1, IL-6), which are similarly dysregulated in both IBD and CRC [25,26,27,28]. While dietary factors have clear effects on organs of the intestinal tract, absorption of these factors results in systemic effects on a number of organ systems, which have been implicated in the progression of a variety of cancers, as detailed in later sections (Figure 1).

In this review we describe some of the key findings supported by peer-reviewed research studies to date regarding cancer and diet, including the impact of dietary factors such as macronutrients, micronutrients, preservatives/additives, and vitamins, and analyse the more recently recognized contribution of the microbiome. We use some examples to elucidate how diet can impact cancer, through the microbiome, and discuss common misconceptions regarding diet and cancer. The review first presents current knowledge on dietary components that have defined (or at least well-supported) action on cancer biology. We then describe some key cancers with strong data on the involvement of dietary factors in the development of malignancy and provide a few examples of how this knowledge could be used to prevent or reduce risk for these cancers. Finally, we highlight the role of microbes in these pathways and suggest potential novel ideas on how dietary or microbe-altering treatments may be used in the future to prevent or even treat cancer, mostly as an adjuvant therapy. While the field of diet and microbes in cancer is not yet supported by a large number of empirical studies, we expect that it will grow over the next few years and will offer new facets to the world of cancer research and patient care. However, given the breadth of this topic, the long list of dietary factors known to impact cancer, and the recent explosion in the field, this review cannot provide a comprehensive list of all relevant dietary components and studies, but rather provides a flavour of how this field has developed (especially over the last couple of years) and what implications this may have.

## 2. Impact of Select Dietary Factors on Cancer Biology

While diet is considered an environmental exposure, it is difficult to measure accurately in humans for several reasons. In contrast to cigarette smoking, for example, which can be expressed as pack-years, food contains thousands of molecules, is not easily quantified, varies geographically, and humans are notoriously inaccurate in reporting consumption [29]. Although it would probably be best to define dietary exposures using food patterns [30], most current research has focused on specific nutrients. A series of recent review articles highlighting the studies examining cancer in relation to specific dietary patterns labelled Mediterranean [31], Western, Ketogenic [32], or other such common dietary patterns [33], demonstrate that the nutrients or food groups that are specific to these diets appear to underscore potential mechanisms behind the positive or negative effects correlated with these diets. This section will present some examples of links between these specific nutrients and cancer (Figure 1).

### 2.1. Protective Effects of Vitamins

Vitamins A, C, and D have been demonstrated to play an anticarcinogenic role in a range of studies [34]. A recent meta-analysis of random controlled trials concluded that Vitamin D significantly reduced mortality (13%) but did not have an impact on cancer incidence, with the exception of colorectal and ovarian cancers [35]. Furthermore, vitamin A has been shown to play a significant role in cell proliferation and differentiation [36] and studies demonstrate an inverse relationship between vitamin A and bladder, colorectal, and liver cancers [37,38,39]. These studies suggest that the effects of vitamins may occur through enhanced DNA repair, antioxidant effects, or immunomodulation of host cells [40,41]. The in-depth role of vitamins has previously been well reported in relation to cancer [42,43].

### 2.2. Sodium and Potassium

Sodium and potassium have long been thought to play a role in the development of cancer as, for example, patients with decreased potassium levels, or hypokalemia (<3.5 mmol/L), associated with cell aging, obesity, alcoholism, and stress, display increased rates of cancer; in contrast patients with hyperkalemia (>5.3 mmol/L) and diseases associated with increased potassium levels, such as Parkinson and Addison disease, display reduced cancer rates [34]. The concentrations of sodium and potassium also interestingly play a role in regulating the effects of calcium, resulting in a series of varied results in studies investigating the tumorigenicity of calcium [34]. Furthermore, almost 50% of cancer patients have been shown to display hyponatremia (<135 mmol/L sodium), although this may also be secondary to the cancer itself [44].

### 2.3. Food Preservatives Linked to Cancer

The sodium salt of propionic acid, sodium propionate, is often used as a food preservative in bakery products and has been shown to exert an antitumour effect through the mitogen-associated protein kinase (MAPK) signalling pathway in breast cancer xenograft models. [45] This example illustrates the complexity between food preservatives, microbes, and cancer, as most are observed to be negatively associated. Nitrates have also been well-described to be associated with colorectal cancer [46], while sorbate and benzoate correlate with the occurrence of breast cancer [47]. Processed foods and the use of food preservatives have dramatically increased over the last few decades and many have attempted to link this with increases in cancer [48]. Food preservatives are a diverse group of ingredients and therefore we have decided to only highlight a few in this review. Most studies focus on negative associations between preservatives and cancer, but to balance the literature, we choose to describe here some studies that display protective effects.

### 2.4. Not All Additives Are Bad: Potentially Beneficial Chemical Food Components

Capsaicin has long been thought to display anti-inflammatory, antioxidant, anti-proliferative, metabolic, and cardioprotective effects and, interestingly, has been shown to affect microbes within the gut too [49]. Decursin, an extract from the *Angelica gigas* root, has been shown to display potent anticancer activity in cell line models of lung and colon cancer, along with Lewis lung carcinoma allograft mouse models of tumour growth [50]. A recent study has shown the capacity of decursin to promote HIF-1α degradation within the proteasome, therefore improving T cell activation and antitumour effects within the tumour microenvironment [50].

A subset of dietary fibres, collectively known as β-glucans, are found in a variety of food groups, from mushrooms and other fungi, to wheat, oats, and barley. Interestingly, raw and roasted barley rich in β-glucan has been shown to provide chemoprotective effects via inhibition of growth and promotion of apoptotic pathways [51]. Similar studies examining the effects of raw and roasted oat flakes, following fermentation with human fecal microbes, demonstrated that the fermentation supernatants, with reduce pH and increased butyrate, significantly decreased growth and increased apoptosis of colon adenoma cells [52]. These findings suggest that food products, mostly made of specific grain β-glucans, harbour chemoprotective potential.

The flower buds of adaptogenic plants, typically found in Chinese traditional medicines, including *Gardenia jasminoides*, *Sophorae japonica*, and *Lonicerae japonicae*, demonstrated a significant effect on reducing polyp burden, along with lowering expression of oncogenic signaling molecules in mice [53]. These changes were associated with a reduction in pathobiont microbes including *Helicobacter pylori,* along with an increase in beneficial microbes, including the key short chain fatty acid (SCFA) producers, *Akkermansia, Barnesiella, Coprococcus, Lachnoclostridium*, and *Ruminococcus* [53]. A diet high in seaweed has been linked with promotion of *Bacteroides plebeius*, which is involved in the breakdown of the seaweed *Sargassum wightii* to polysaccharides (SWP1 and SWP2); this significantly reduces cell proliferation and induces apoptosis in human breast cancer cell lines [54,55]. Seaweeds’ health benefits have been associated with the polyphenols, polysaccharides, sterols, and bioactive molecules that have been shown to play a role in anti-inflammatory and anticancer effects [56].

## 3. Microbes as Key Mediators in Diet−Cancer Interactions

The gut microbiome is known to have significant consequences on human health and disease, including cancer, as demonstrated by studies where direct modification of gut microbes, through fecal microbial transplantation, for example, impacts cancer outcomes and response to therapy [57]. We focus in this section on an indirect role of microbes, which likely provide a link between diet and cancer, and have highlighted some key recent examples in Table 1. However, there is still a knowledge gap between changes in the microbiome due to diet and long-term consequences, such as cancer development. A recent study looking at the effects of ketogenic diet on the microbiome found a reduction in *Bifidobacterium* sp., which are known to increase gut Th17 cells, together with increases in *Fusobacterium* and *Escherichia* through the production of ketone bodies [58]. While evidence supports the fact that a ketogenic diet decreases long term health due to high consumption of dietary fats, limited studies have demonstrated that specifically when combined with select cancer therapies, a ketogenic diet may improve outcomes for those suffering from cancers involving the protein kinase B (AKT)/mechanistic target of rapamycin (mTOR) pathway [59]. *Fusobacterium* and Th17 cells are also associated with an increased risk of colorectal cancer, suggesting that ketogenic diets may be detrimental when used long-term [60]. A diet high in fats or meats has also been shown to increase the abundance of select opportunistic microbe species responsible for the production of enzymes such as B-glucuronidase, which plays a key role in xenobiotic-induced toxicity within the intestine [61,62]. B-glucuronidase has been previously associated with increased risk of select breast cancers [63], pancreatic cancer [64], and colorectal cancer [65], and recent evidence supports the use of inhibitor compounds targeting B-glucuronidase for improved anticancer efficacy [66].

There are few studies looking at the effects of food preservatives on the microbiota, typically limited to using animal models [67]. A recent study showed that a combination of antimicrobial food preservatives (sodium benzoate, sodium nitrite, and potassium sorbate) induced a decrease in the abundance of Firmicutes (*Clostridiales* order) while increasing Proteobacteria (*Burkholderiales* order) in human-microbiota associated mice, suggesting a possible link to dysbiosis [68]. A recent literature review illustrated evidence suggesting that food additives can drive dysbiosis through the reduction of anti-inflammatory bacteria such as *Clostridium tyrobutyricum* and *Lactobacillus paracasei* with a link to irritable bowel syndrome [69].

Interestingly, studies have demonstrated variable effects of the Na/K ratio on microbe diversity, based on geographical location (environment), suggesting that sodium and potassium consumption play a variable role in regulating the abundance of pathogenic microbes, such as *Staphylococcus* and *Moraxellaceae*, along with SCFA-producing microbes, such as *Phascolarctobacterium* and *Lachnospiraceae* [70]. Specifically, studies suggest that increased sodium consumption results in decreases in key microbial metabolites, including butyrate and isobutyrate, along with anti-inflammatory phenols [70,71].

A recent review article clearly outlined the diet–microbe interaction with regards to prostate and colon cancers [72]. This review summarized studies demonstrating that an increase in dietary fibre may provide the necessary nutrients for the establishment of the microbiota involved in butyrate production, leading to improved colorectal cancer outcomes. The authors suggest a link between linoleic acid (found in high fat diets) and prostate cancer, explained by cancer cells having a higher concentration of arachidonic acid and prostaglandin E2. Linoleic acid metabolic pathway has also been linked to colorectal cancer progression in individuals with ulcerative colitis combined with enrichment of *Enterobacteriaceae* and Proteobacteria [73].

Although the intestinal microbiota has profound effects on our overall health, it is important to also consider the oral microbiota, which is clearly impacted by diet. Periodontitis is a microbial-induced chronic inflammatory disease associated with dysbiosis of the oral microbiota that can lead to systemic health issues, such as type 2 diabetes [74,75]. The oral microbe *Fusobacterium nucleatum* is increased in patients with periodontitis, which is also associated with an increased risk of colorectal cancer [75,76]. In addition, a recent study showed that individuals with pancreatic cancer have higher circulating and salivary antibodies against *F. nucleatum*, suggesting a link between oral microbiota and pancreatic cancer [77]. Polyphenols are first metabolized in the oral cavity, modulating inflammatory responses as well as eliciting antimicrobial activity, inhibiting the growth of *F. nucleatum* [76]. Although the metabolism of polyphenols in the oral cavity is largely unknown, data suggest that diet also modulates the oral microbiota and therefore impacting our overall health. In addition, polyphenols from foods such as red grape wine, cocoa, and blueberries have anti-inflammatory and antioxidant properties that have potential for prevention of other cancers, such as colorectal cancer [76]. A recent review discussing the in-depth role of polyphenols in cancer has brought new light to this topic [78].

More recent research has highlighted the role of the local microbiome of the tumour microenvironments outside of the gut; for example, a Mediterranean diet has been shown to alter the microbiome of the breast by increasing the abundance of microbes, such as *Lactobacillus*, when compared to a western diet [79]. Further, studies have begun to identify a series of distinct tumour-specific microbiomes associated with select cancers [80,81,82], which can be associated with therapeutic response [83,84]. Understanding these distinct microbe profiles may allow for early detection in the future, or allow for preventative measures through microbe-altering therapies or dietary interventions.

### 3.1. Microbe-Altering Approaches to Cancer

Although changes in the microbiota can be detrimental, there may be some microbe-altering approaches that may be used in the treatment or prevention of cancer. Studies on the effects of probiotic use have largely focused on colon cancer. There is evidence that the probiotic *Clostridium butyicum* may inhibit intestinal tumour development through suppression of the Wnt/ß-Catenin pathway as well as modulation of the gut microbiota [85]. The Wnt signalling pathway has a central role in stimulating and promoting cell proliferation; mutations in the Wnt pathways are frequently found in carcinomas, specifically CRC [86]. Probiotics have the potential to influence cell signalling pathways in the intestinal tract, however more studies are needed to determine if they indeed have a role in CRC and whether they are beneficial for other cancers.

### 3.2. SCFA from Microbial Metabolism

Fermentation of nondigestible carbohydrates (starch and fiber) and proteins by select microbes results in the production of SCFAs [87,88]. A variety of dietary components associated with SCFA production have been shown to further improve response to cancer therapies, including immune checkpoint blockers, as highlighted in a recent review by Russo et al. [89]. A number of SCFAs have been shown to significantly inhibit proliferation of cancer cells in vitro. This has been demonstrated by modulating multiple signalling pathways, including the inhibition of histone deacetylase (HDAC), inducing apoptosis, and upregulating select G protein-coupled receptors (GPCRs/GPR) associated with cancers [90]. Interestingly, omega-3 polyunsaturated fatty acids (PUFAs), commonly found in fish oils, have also been demonstrated to play a role as agonists of these GPRs, namely GPR40 and GPR120, as documented in a recent review by Freitas and Campos [91]. Furthermore, n-3 PUFAs have also been shown to modulate the intestinal microbiome, leading to improved intestinal barrier integrity and reduced inflammation [92,93,94].

Of the SCFAs, propionate has been shown to upregulate the immune stimulatory and antitumorigenic NKG2D ligand, MICA/B, through pathways not directly associated with the traditional SCFA receptors GPR41/43 [95,96]. Propionate further participates as a precursor molecule for acyl-CoAs that are involved in the acetylation of histones, which reduces cancer phenotype and improves clinical outcomes [97]. The SCFAs acetate, butyrate, and propionate have been further shown to promote IL-22 production, specifically in CD4^+^ T cells and innate lymphoid cells, through elevation of hypoxia-inducible factor (HIF)1α, interaction with GPR41, and inhibition of HDAC [98].

The microbe *Butyricicoccus pullicaecorum* produces the SCFA butyrate, which activates select SCFA transporter (SLC5A8) and receptor (GPR43) pairs, improving the clinical outcome of a colorectal cancer model in mice [99]. Butyrate specifically decreases intercellular adhesion molecule (ICAM)-1, which plays a key role in leukocyte migration in the oral epithelium; this decrease results in reduced inflammation and may play a protective role in progression to oral squamous cell carcinoma [100]. A recent review by Hajjar et al. nicely highlights the evidence to date which demonstrates that butyrate’s natural anticancer activity is in part modulated by its effects as a HDAC inhibitor [101], suggesting supplementation of butyrate in patients as a potential antitumour therapy. Sodium butyrate has further been shown to induce growth arrest and inhibit DNA synthesis in breast, prostate, and colorectal cancer [102,103,104,105,106]. Sodium phenylbutyrate, which is rapidly metabolized to the metabolically-active phenylacetate, has been examined in both clinical and preclinical settings, demonstrating positive effects when combined with chemotherapeutics, cisplatin, gefitinib, or erlotinib, to promote apoptosis in pancreatic, cervical, colon, and central nervous system cancers [107,108,109,110]. Increased systemic butyrate and propionate, thought to be due to increased intestinal permeability, have typically been associated with immune modulation via induction of Treg cells, and recently have been shown to limit antitumour activity of anticytotoxic t-lymphocyte-associated protein 4 in multiple tumour mouse models [111].

Interestingly, studies have demonstrated key microbial changes in premenopausal patients at time of breast cancer diagnosis, suggesting that specifically SCFA-producing microbes are significantly lower in abundance in premenopausal breast cancer patients [112]. Furthermore, the SCFAs propionate and butyrate have been shown to inhibit breast cancer cell growth in vitro [112]. Valproic acid, a histone deacetylase inhibitor, has recently been demonstrated to inhibit growth of glioma cells in models of high-grade gliomas [113]. In contrast to the highlighted positive effects of SCFAs, the bacterial metabolite butyric acid plays a role in exacerbating ameloblastoma, a benign tumour of the jawbone, through interactions with epidermal growth factor and transforming growth factor β1 secreted by the tumour cells [114].

## 4. Examples of Cancer Impacted by Diet, Linked to Microbiome

**Figure 2:**
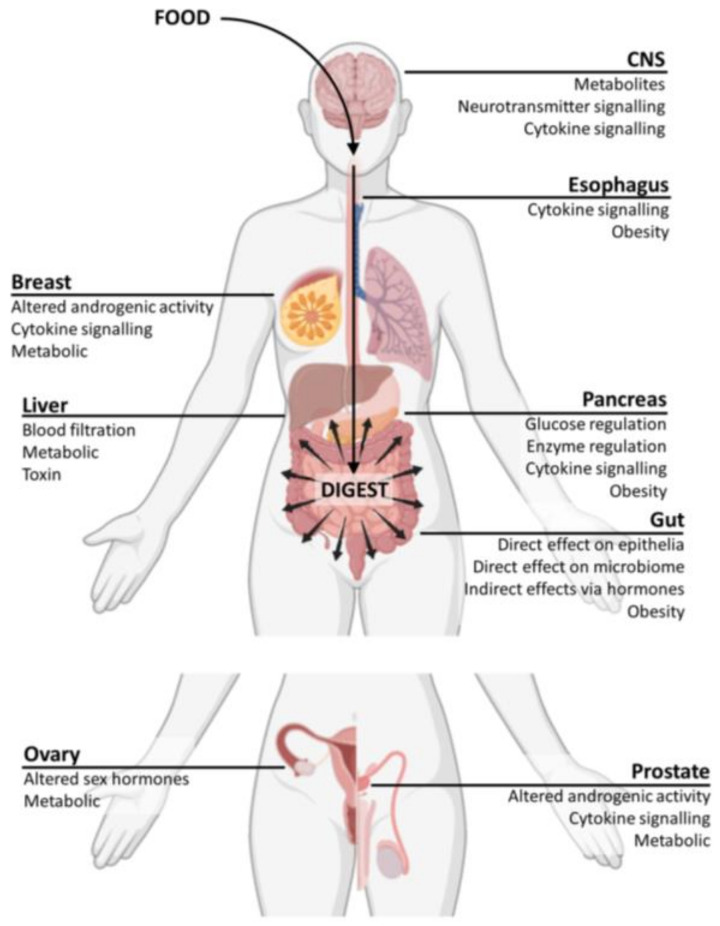
Potential effects of dietary factors on organs effected by cancer. Intake of dietary factors potentially affect bodily organs through direct filtration of blood or absorptive processes, or indirectly through regulation of metabolites absorbed by the gut, glycemic control (glucose, insulin, cholesterol), regulation of metabolic pathways, inflammatory response to systemic cytokine production, altered sex hormone activity, neurotransmitter metabolism, and response to chronic obesity, as indicated.

Diet clearly has an impact on every aspect of human health and disease. One of the biggest challenges is that diet is difficult to measure, dietary interventions are hard to enforce, and documenting adherence is known to be biased. Still, there are some examples of cancers with quite strong support for a role for diet. Some of these key conditions are detailed in the text below and in Table 1, while the potential mechanism of interaction between dietary factors and select organ systems will be highlighted below (Figure 2).

**Table 1:** Examples of recent links between cancer, microbes, and dietary factors.

Cancer Type	Possible Microbes Involved	Dietary Risk Factors	Preventative Dietary Factor
CNS, Neuroblastoma and Glioma	*Bifidobacterium adolescentis* [58]		Valproic acid [113] Ketogenic diet [115,116,117] Sodium phenylbutyrate [107,108,110]
Esophageal	*Pasteurellales* [118]	Alcohol consumption and high temperature beverages [119]	Fruit and vegetable [120] Polyphenols [76,100]
Breast	*Bacteroides plebeius* [54,55]	Acid-producing diets [121,122] B-glucuronidase [63]	Valproic acid [123,124] Seaweed [54,55] Sodium propionate [45] Sodium butyrate [102,103,104,105,106] Mediterranean diet [112,123,124] Sorbate [47] Benzoate [47]
Ovarian	*Clostridia* [125]		Valproic acid [90] Vitamin D [35]
Prostate	Possibly *Enterobacteriaceae* and *Proteobacteria* [73]	High-fat (keto) diet [72]	SCFA [72,126] Sodium phenylbutyrate [107,108,110] Sodium butyrate [102,103,104,105,106]
Hepatocellular carcinoma	*Streptococcus*, *Bifidobacterium*, *Enterobacter* and *Atopobium* [127]	Alcohol and red meat [21,128]	SCFA [129,130] White meat [128] Vegetables [128] Fruit [128] Vitamin A [37,38,39]
Pancreatic	*Fusobacterium nucleatum* [77]	High-fat (keto) diet [58,77,131] B-glucuronidase [64]	SCFA [132,133] Sodium phenylbutyrate [107,108,110]
Colorectal	*Fusobacterium nucleatum* [58] *Clostridium butyricum, Butyricicoccus pullicaecorum* *Akkermansia, Barnesiella, Coprococcus, Lachnoclostridium*, and *Ruminococcus* [53,85,99]	High-fat (keto) diet [58,60,73] Red meat [21] B-glucuronidase [65]	SCFA [70,72,99] Probiotics [85,86] Decursin [50] Sodium butyrate [85,99,102,103,104,105,106] Sodium phenylbutyrate [107,108,110] Vitamin D [35] Vitamin A [37,39] Nitrates [46] Oat flakes [52] Adaptogenic flower buds [53] n-3 PUFAs [91] Polyphenols [76,78]

### 4.1. Neuroblastoma and Gliomas

Cancers of the brain such as neuroblastoma, which develops during nervous system expansion in the embryo, or glioma, developing in the adult postmitotic brain, represent rare, yet highly lethal forms of cancer [134,135]. Limited evidence has supported a role for diets such as the ketogenic diet in reducing tumour burden in neuroblastoma xenograft models [115]. Ketogenic diets have recently been shown to reduce proliferation and stemness in glioma cells through metabolic changes that result in a disproportional increase in reactive oxygen species (ROS) production associated with cell arrest and apoptosis [116,117]. Intake of dietary factors potentially results in effects on the brain through regulation of metabolites from the gut-driving glycemic control (glucose, insulin, cholesterol, SCFA), systemic cytokines altering inflammatory activity, and neurotransmitter metabolism [136,137].

### 4.2. Esophageal Cancer

A number of risk factors have been associated with esophageal cancers, especially the more prevalent esophageal squamous cell carcinoma, including smoking, alcohol consumption, consumption of high temperature beverages, genetics, and diet [119]. Specifically, diet appears to have both direct and indirect effects on the esophagus through cytokine signalling pathways and obesity [138,139,140].

### 4.3. Breast Cancer

Breast cancer remains the most common malignancy among women, at times treated using the antimetabolite capecitabine, which, in combination with SCFA, has been shown to provide even greater antitumour effects [90]. SCFAs, namely valproic acid, provide proapoptotic and anti-proliferative effects in breast cancer cells [123,124]. Foods that would promote production of these microbial metabolites could be of benefit in this setting, while others have been shown to function through altering androgenic activity, regulating metabolism, and through cytokine signalling [141].

### 4.4. Ovarian Cancer

The effects of diet in ovarian cancer are thought to operate through similar pathways to breast cancer, via altered sex hormones and regulation of metabolism [141]. Most interestingly, SCFA have been demonstrated to play a role in inhibiting HDAC in ovarian cancer cells lines and it has been suggested that combining valproic acid with chemotherapeutics such as paclitaxel or doxorubicin could result in increased apoptosis, decreased poly (ADP-ribose) polymerase enzymes, and inhibition of DNA repair in ovarian cancer cells, although yet to be tested clinically [90].

### 4.5. Prostate Cancer

Similarly, SCFAs have been shown to target histone acetylation processes resulting in the re-expression of cyclin D2, which results in the inhibition of prostate cancer cell growth, migration, and invasion [126]. Dietary factors are thought to affect the prostate via altered androgenic activity, cytokine signalling pathways, and through metabolic effects [142,143,144].

### 4.6. Hepatocellular Carcinoma (HCC)

HCC progression has been directly associated with increased Notch signalling, which is downregulated by microbe fermentation processes and in response to SCFAs [129,130]. The specific interactions between the liver and dietary factors are thought to occur through blood filtration of absorbed nutrients, toxins, and metabolites, as well as thorough systemic metabolic regulation [128,145].

### 4.7. Pancreatic Cancer

Some of the most exciting evidence on the use of diet and microbial strategies in cancer relates to pancreatic cancers. Phase I and phase II clinical trials have been performed based on the positive effects of SCFAs as antitumour aids in pancreatic cancer [132,133]. These studies demonstrated 91.7% tumour control in patients with advanced stage pancreatic cancer [132]. The pancreas plays a well-known role in the regulation of metabolic pathways and therefore it is not surprising that its interactions with dietary factors occur through glucose and enzyme regulation, cytokine signalling, and indirectly through the effects of chronic obesity [146].

### 4.8. Colorectal Cancer

Colorectal cancer represents one of the leading causes of cancer-related death, which is thought to be due to dietary changes, increased incidence of chronic IBD, and increasing life expectancies globally. Diet has been suggested to play a leading role in up to 50% of colorectal cancer cases and probiotics have shown some benefit in the treatment of IBD, diarrhea, irritable bowel syndrome, gastroenteritis, and cancer, suggesting that altering diet and microbes could be an effective therapeutic option for colorectal cancers [147]. However, clinical data on the utility of diet and microbe-related treatments for CRC are still in the early stages. The gut is the primary site of dietary interactions within the body as it is responsible for the majority of the digestive and absorptive processes resulting in direct effects within the epithelial lining, alterations of the host microbiome, metabolic regulation, immune response, systemic hormone alterations, and indirect responses related to chronic obesity [148,149].

## 5. Common Misconceptions Regarding Diet and Cancer

As research continues to demonstrate the powerful links between diet, microbes, and progression to cancer, it is important to distinguish what facts are supported by science, on the one hand, and the myths that continue to plague popular belief, on the other. We recently reviewed a number of common misconceptions in relation to diet, microbiome, and cancer [6] and will update and expand on these ideas here, relevant to this review.

One common misconception involves the idea that consumption of organic foods reduces risk of cancer. In a decade-long study of 623,080 middle-aged women in the UK, researchers demonstrated that there was little to no decrease in risk of cancer associated with an organic diet [150]. Interestingly, one French study of 68,946 participants followed over seven years, suggested that heightened frequency of organic food consumption was in fact associated with reduced risk of cancer; however, one major drawback of this study that is commonly overlooked, pertains to the fact that the occurrence of cancer also correlated with the overall consumption of fiber, fruits, and vegetables, and their findings were not adjusted to these residual cofactors [151]. A number of studies have demonstrated the protective correlation between a diet high in fruits and vegetables [120,152,153,154,155,156,157] resulting in a lack of substantiated reason to suggest that organic food consumption holds any protective benefits.

Although not well supported at this time, one possibility that may explain the connection between select organic foods and cancer risk includes the consumption of pesticides [151,158,159,160]; however, a recent publication highlighted the serious limitations within the studies of dietary pesticide consumption and the connection to cancer risk resulting again, in a lack of substantiated reason to suggest that organic food consumption holds any protective benefits [161]. Once more, the likely reason behind the results of this particular study relates to lifestyle and demographic covariates as this was not an interventional study but rather observational. On this topic, it is also important to note the large geographical and regulatory differences in pesticide use globally, leading to variability in how we may relate study findings to outcomes [162].

Alkaline diets are often misconstrued to be protective of development of cancer, although there currently remains little research to support or disprove this concept [163]. Recent evidence from two observational studies suggests that diets categorized as acid-producing are associated with greater risk of inflammation in estrogen receptor-negative and triple-negative breast cancers [121,122]; these studies support the need for ongoing research on this topic.

One interesting highly popularized misconception that crosses multiple fields of health research is that of cleansing or detoxes for use in “removing toxins”, harmful substances, or cancer cells from the body. The body has a series of organs (liver and kidneys) and natural processes in place to complete these tasks and there remains no evidence to date for any magic potion to replace these processes; in fact, the harmful effects on fluid balance and loss of nutrients need to be considered [164,165,166,167,168,169]. At this time, we would promote general recommendations that individuals avoid heavy consumption of alcohol and eat a healthy, well balanced diet, to ensure proper organ function continues.

In contrast to those common misconceptions that involve dietary factors that are not in fact associated with cancer risk, the subject of sugar consumption in relation to cancer is a difficult topic to summarize and is commonly misconceptualised as “unassociated” with cancer. There are a number of reasons for this, namely that all of the cells in the human body utilise sugars as an energy source and therefore require sugars for proper organ function [170]. That being said, many studies have correlated the outcomes of a poor diet, high in sugars, with health conditions (e.g., obesity) that are positively correlated with increased risk of cancer [171,172]. Within recent years a number of published studies have demonstrated that high glucose both in vitro and in clinical settings not only drives promotion of cancer, but also represses the anti-proliferative and pro-apoptotic effects of cancer therapies, including Metformin [171,173,174]. While avoiding consumption of sugar altogether is not necessarily beneficial, it should be recommended that a high-sugar diet be avoided to reduce risk of cancer, and to prevent interference with select anticancer therapies.

Misconception and myths continue to plague the field of medicine surrounding the concepts of diet and cancer; however, it is important to recognize the pros and cons, and potential risks involved. Ongoing research will continue to uncover new beneficial and detrimental factors associated with cancer; until then, researchers will need to ensure that fact-based evidence is promoted over misconception and myth.

## 6. Future Perspectives and Opportunities

Cancer remains one of the most prevalent diseases in the western world, and a leading cause of death, yet our comprehension of the role of diet and microbes in promoting inflammation and malignancy remains limited. As research continues to demonstrate, providing clinical advice related to nutrition and microbiome can be difficult for a number of reasons, including the limited understanding of these topics to date, in part due to the complexity of these studies. Here we discussed some key recently published studies, which have accelerated our understanding of dietary factors and microbes in a number of cancers.

We recognize that there are a number of complex systemic factors related to cancer development, progression, and prevention that were not thoroughly covered in this review and would like to highlight selective recent papers and reviews related to some of these aspects. For example, hormones appear to impact the interaction between diet, the microbiome, and cancer, as demonstrated by comparing the gut metagenome in pre-menopausal and post-menopausal women, with and without breast cancer [175], and reviewed elsewhere [176]. Ketogenic diets have been discussed here in the context of glioblastoma, prostate, pancreatic, and colorectal cancers, but are likely relevant in a broader sense to other settings, as the tumour microenvironment created with this diet has the potential to suppress or promote cancer cells, and regulate the effect of antitumour therapies [131,177], dependent on the individual’s microbiome [178]. Another important aspect connecting the diet–microbiome axis to cancer is epigenetics, as DNA methylation and histone modification, including HDACs, as highlighted in this review, are impacted by dietary and microbial compounds, with a potential to mediate additional mechanisms leading to cancer [179]. With improving technologies (high-throughput sequencing and omics [180,181]) and evolving methodologies for tracking dietary intake (openly available rapid and accurate reporting tools [182,183]), we may continue to improve the significance of ongoing research associating microbes and diet with cancer.

By combining the data published to date, one begins to notice patterns associated with the benefits of consumption of fruits, vegetables, and grains made up of fermentable dietary factors that are utilised by beneficial microbes, ultimately resulting in the production of key SCFAs and reduced risk of malignancy. Similar findings have been established in studies of a broad range of diseases, suggesting that consumption of greater amounts of these foods, in combination with the appropriate microbes, imparts beneficial health effects. These studies do not necessarily promote a vegetarian diet, so much as a diverse diet high in fruits and vegetables, yet the studies to date do support limiting the consumption of red meats [21] in particular. It is imperative that we consider that it is in part the gut microbes that require the correct nutrients, sourced from dietary intake, in order to survive, thrive, and aid in promoting host health; dysbiosis, or loss of key microbes, is associated with a number of chronic illnesses that are associated with increased cancer burden.

One of the biggest challenges in determining the involvement of microbes and diet in health is the need for long term studies, performed over decades, which examine precise metabolites (metabolomics), microbiome (sequencing), epidemiology, dietary intakes, and clinical outcomes. By ensuring only the most precise and up-to-date techniques are utilised in future studies, using thorough planning and preparation, we can ensure the combination of basic, translational, and clinical research outcomes are able to direct precision medicine involving microbe-altering approaches and dietary interventions reducing cancer burden. A number of fantastic reviews [181,183] have recently highlighted the best techniques in use at this time, and further review should be performed during any planning phase in order to ensure the appropriate techniques are being utilised to ensure translation of results for clinical use.
